# Antimicrobial use in 20 U.S. beef feedyards: 2018–2019

**DOI:** 10.3389/fvets.2023.1056362

**Published:** 2023-03-27

**Authors:** Michael D. Apley, Nora F. D. Schrag, David E. Amrine, Brian V. Lubbers, Randall S. Singer

**Affiliations:** ^1^Department of Clinical Sciences, College of Veterinary Medicine, Kansas State University, Manhattan, KS, United States; ^2^Livestock Veterinary Resources, LLC, Olsburg, KS, United States; ^3^Department of Veterinary and Biomedical Sciences, College of Veterinary Medicine, University of Minnesota, St. Paul, MN, United States; ^4^Mindwalk Consulting Group, LLC, Falcon Heights, MN, United States

**Keywords:** antimicrobial use, feedyards, feedlots, antimicrobial use monitoring, antimicrobial use reporting, antimicrobial indications, antimicrobial outcomes

## Abstract

The objective of this study was to report antimicrobial use in a convenience sample of U.S. beef feedyards for the years 2018 and 2019. In addition to antimicrobial use metrics, also reported are the indications for antimicrobial use and outcomes related to these indications. Antimicrobial use is characterized at the study and feedyard levels for a total of 1,141,846 head of cattle in 20 U.S. feedyards. Antimicrobial use is reported as milligrams of active antimicrobial ingredient per kilogram of liveweight sold (mg/kg-LW) and regimens of antimicrobials per animal year (Reg/AY). Regimens are described by antimicrobial class within use category as characterized by mg of active antimicrobial product per regimen (mg/Reg) and calendar days of administration per regimen (CDoA/Reg). A total of 1,128,515 regimens of medically important antimicrobials were captured from records. The number of regimens/100 head-in (Reg/100 head-in) are described in a subset of 10 feedyards with adequate data granularity to directly determine indications for antimicrobial administration. For the indications of bovine respiratory disease (BRD), Lameness (Lame), Liver Abscess Control (LAC), and Other (e.g., central nervous system disease, cellulitis) the Reg/100 head-in study-level values are 37.1, 0.8, 98.4, and 0.7, respectively, for 2018, with similar values for 2019. The regimens for BRD are further categorized in these 10 feedyards by the use categories in-feed, control of BRD, and individual animal therapy, yielding study level values of 4.6, 19.6, and 12.9 Reg/100 head-in, respectively, for 2018, with similar values for 2019. Outcomes of therapy for individual animal treatment of BRD, Lame, and Other are reported as treatment success, retreatment, or mortality by 30 days after the initial therapy of an animal for a disease. Treatment success rates (no treatment or mortality in the next 30 days) for 2018 in the 10 feedyards with sufficient data granularity are 76.5, 86.5, and 83.0% for BRD, Lame, and Other, respectively. The comparison of these results with other reports of antimicrobial use in North American feedyards highlights how differing approaches in calculating metric values may result in substantially different conclusions regarding antimicrobial use, especially in relation to long-duration uses.

## 1. Introduction

In 2016, the FDA Center for Veterinary Medicine funded two cooperative agreements to collect data from food animal production facilities in the United States with the goal of evaluating the potential for collecting more granular antimicrobial use data in U.S. food animal production systems. One of the cooperative agreements was focused on swine and poultry with data from the first period of data collection recently published (1–3). The other cooperative agreement was focused on beef feedyards and dairies, also with initial data and analysis for the years 2016 and 2017 being published (4–8). This publication constitutes reporting of antimicrobial use data from 20 U.S. beef feedyards for a subsequent period of data collection during 2018 and 2019. In addition to antimicrobial use reporting similar to the previous publications, the results reported here also include characterization of antimicrobial use indications linked directly to the regimens, and reporting of treatment outcomes for these disease-regimen pairings.

This paper reflects the worldwide efforts in antimicrobial stewardship in both human and veterinary applications through an important initial step of characterizing use. In the United States, at the time of this writing the FDA has commissioned the Reagan-Udall Foundation to explore the potential for creating a public-private partnership to move forward with antimicrobial use monitoring in food producing animals. This process is at the stage of an initial report with more work in progress (9). An important component of these efforts to establish a national antimicrobial use monitoring program is to agree on standard antimicrobial use metrics and how they are to be calculated. Another vital component is linking antimicrobial use to both reason for use and the outcomes for these uses. As reported here, the authors link antimicrobial use not only to the indication for use, but also outcomes. While these outcomes may not be considered as an indication of antimicrobial efficacy due to lacking appropriate controls, they are able to put the antimicrobial use in a context of disease effects. Consideration of the implications of different decisions in beef feedyard antimicrobial use monitoring programs is presented in the discussion through comparing the results of this studies to several other efforts to characterize antimicrobial use in North American feedyards. The reader is referred to a companion paper for an evaluation of the characteristics of multiple antimicrobial use metrics in feedyards (10).

The authors emphasize that the feedyards in this study represent a sample of convenience and should not be interpreted as a random sample of the industry which represents industry-wide practices.

## 2. Materials and methods

### 2.1. Feedyard description

The previously reported data for 2016–2017 were collected from 22 beef feedyards (5). In this report of data from 2018 to 2019, 19 of the feedyards are the same, with 1 of the previous feedyards withdrawing, 1 ceasing to feed cattle, and 1 altering its record system use in a way that prevented standardization. One feedyard which was approached for the initial round of reporting but was unable to provide appropriate feed antimicrobial inclusion data at that time was now able to do so and was added to the project. These attritions and addition resulted in a total of 20 feedyards represented in the data reported here. These changes in participating feedyards consisted of smaller feedyards with smaller effects on study level metric values, but temporal trends should still be interpreted with caution.

Participating feedyards were located in Colorado, Iowa, Kansas, Nebraska, and Texas. Data confidentiality was a condition of the investigators having access to the data, which was provided either directly from the cooperating feedyard or through their data software provider. The distributions of cattle sold by participating feedyards in 2018 and 2019 are reported in Table 1. These values represent the lots of cattle for which data met quality standards and were included in analysis; the actual number of cattle closed out for individual feedyards was higher. Cattle included in the analysis for 2018 and 2019 were 565,404 and 576,442, respectively, for a total of 1,141,846. Reported steer and heifer slaughter by the United States Department of Agriculture for 2018 and 2019, and percent of these totals represented by cattle in this study, were 25,803,500 (2.19%) and 26,116,700 (2.21%), respectively.

**Table 1:** Description of participating feedlots.

	**2018**	**2019**
Number of cattle sold	565,404	576,442
**Stratification by number sold**		
≤2,500	3	2
2,501–5,000	1	3
5,001–10,000	2	2
10,001–25,000	7	4
25,001–50,000	4	5
50,001–75,000	1	2
75,001–100,000	1	1
>100,000	1	1

### 2.2. Description of record systems

A variety of record system types were encountered in this second period of data collection as presented in Table 2. There were 3 commercially available electronic records systems used by cooperating feedyards, along with custom electronic systems. One feedyard transitioned from one electronic system to another midway through year 2018. In some cases it was necessary to evaluate manually recorded data and purchase records for entry into an electronic format.

**Table 2:** Description of record systems.

**Record system**	**# Feedyards**
Electronic system #1 direct or indirect	11
Electronic system #1 combined with custom system	1
Electronic system #3 transitioning to system #2	1
Electronic system #3—manual entry or PDF scan from provided records	2
Custom record system directly or indirectly provided	2
Records for purchase or number treated, some in combination with custom record systems—manual entry	3

Fourteen feedyards provided data in a manner which allowed direct determination of regimens through individual animal drug administration records or lot-level administration records for injectable antimicrobials, and lot-level records for in-feed administration. For the other 6 feedyards, some portion of individually administered and/or in-feed antimicrobial records required assumptions based on defined daily doses or defined course doses to assign the number of regimens to a population as well as the indication for administration, as previously described (5). These defined doses were most commonly calculated based on estimated weight (by feedyard personnel) at the time of treatment and a stated common regimen, which in almost all cases was based on the label dose. This method of determining the number of regimens is referred to as resulting in “constructed” regimens in this paper. Both directly determined and constructed regimens are included in metric totals (mg/kg-LW, Reg/AY), but only the 14 feedyards with directly determined regimens are included in the description of regimens in order to only describe actual recorded regimens.

Ten feedyards provided data of sufficient granularity related to individual animal disease indication, along with complete treatment history and mortality, to allow determination of treatment outcomes for bovine respiratory disease (BRD), lameness, and additional instances of individual animal antimicrobial treatments grouped together as “other”.

### 2.3. Data management

As a point of terminology clarification, the term “lot” refers to a group of cattle for management and economic purposes during their time in a feedyard. A lot may be kept in one or several pens and may move locations in the feedyard during the feeding period. Data are recorded in relation to each lot of cattle; therefore, data analysis begins at the lot level.

This paper uses the terms “use category” and “indication” to categorize the administration of antimicrobials. An antimicrobial regimen is accounted for within both a use category and a disease indication.

#### 2.3.1. Antimicrobial use categories

The 3 use categories focus on how the antimicrobial was administered. (1) In-feed administration includes tylosin for reduction in the incidence of liver abscesses at slaughter and chlortetracycline for the treatment or control of BRD depending on the regimen utilized. (2) Control of BRD is reported separately because antimicrobials in this use category are administered by individual animal injection to a group of cattle, most often in an initial processing situation. (3) Individual animal treatment includes administration of antimicrobials to individual animals in response to a diagnosed disease indication.

#### 2.3.2. Disease indication for use

Disease indication connects antimicrobial use to a reason for use. Disease indications apply when an antimicrobial administration is linked to a specific disease diagnosis. The categories for observed disease indications utilized for this study include bovine respiratory disease (BRD), lameness (lame), and all other recorded disease indications combined (other). In the analysis reported here, antimicrobial administrations without an associated diagnosis are categorized as other. As an example, the BRD indication includes chlortetracycline administered in the feed, antimicrobials administered for control of BRD at processing, and antimicrobials administered individually to cattle for treatment of BRD. Liver abscess control (LAC) is also used as a disease indication for the analysis reported here. The use of tylosin in the feed for reduction in the incidence of liver abscesses is a unique disease indication in that this condition is not diagnosed antemortem in the feedyard, but rather at processing.

#### 2.3.3. Quality control procedures

Quality control procedures were conducted as previously reported, using R (5). Briefly, the initial quality control steps consisted of assuring that data at the lot level were in reasonable ranges for days-on-feed, head-in, in-weight, and out-weight. After this step, each lot was evaluated for having all necessary antimicrobial use data. In this second step, 11.2% (2018) and 12.8% (2019) of the initial head count were excluded from analysis due to inadequate antimicrobial records, primarily due to the inability to match feed consumption records with lots, and therefore determine the in-feed antimicrobial use for those lots. The majority of these issues were in four feedyards.

#### 2.3.4. Reporting of antimicrobial use

Reporting of antimicrobial use is at both the study and feedyard levels using the metrics milligrams of antimicrobial per kilogram of liveweight sold (mg/kg-LW) and antimicrobial regimens per animal year (Reg/AY) reported by calendar year in which the cattle were sold. Values are reported within the use categories of in-feed administration, control of bovine respiratory disease (BRD), and individual animal treatment. All values for numerators (milligrams and number of regimens) and denominators (kilograms of liveweight sold and animal years) were first determined at the lot level where they were stratified by use-category, disease indication, and antimicrobial class. At the study level, the total number of milligrams of an antimicrobial across all lots was divided by the total amount of kilograms of liveweight sold across all lots to calculate study level mg/kg-LW (within calendar year). The total number of regimens for an antimicrobial across all lots was summed and divided by the total animal years across all lots to calculate study level Reg/AY (within calendar year). Feedyards with higher numbers of cattle have a greater effect on the study level values than do smaller feedyards.

When values are reported at the feedyard level, the above process is performed for each feedyard, followed by reporting median and mean (with standard deviation) values across all feedyards being described. At the feedyard level, each feedyard has an equal effect on the values regardless of their size.

The description of regimens includes milligrams per regimen as previously reported (5), but in this paper the previously reported regimen timeframe (days from first administration to last administration) has been replaced with calendar days of administration per regimen (CDoA/Reg). The metric CDoA/Reg describes the counted days of administration of an antimicrobial associated with each regimen.

Describing and counting the regimens for tylosin presented a challenge in 4 feedyards due to a break in continuity for the feeding of tylosin inconsistent with typical feeding practices. This may have been due to discontinuity in the merging of feed inclusion data with feed delivery data, intentional discontinuation of feeding, or supply or logistical issues. To address the issue of potential merging errors, if the calendar days of administration of any one duration of tylosin administration was greater than 20 days, it was considered a separate regimen and the two periods of administration were considered separate regimens for the purpose of quantifying regimens. Using this rule, there were 32,148 additional regimens of tylosin (2.8% of reported tylosin regimens) in the total count where these regimens were in addition to another counted regimen.

Reporting is divided into medically important and not-medically important sections. Classification of antimicrobials as “medically important” or “non-medically important” was as classified in Appendix A of the Food and Drug Administration Center for Veterinary Medicine Guidance Document #152 (11).

## 3. Results

### 3.1. Medically important antimicrobials at the study level

The number of regimens stratified by antimicrobial class within use category are reported in Table 3. The percent of total regimens represented by in-feed administration, control of BRD, and individual animal treatment were 77.8, 12.7, and 9.6%, respectively. Macrolides accounted for 82.0% of all regimens, this total value being comprised of 72.8% of total regimens as tylosin administered in the feed for reduction in the incidence of liver abscesses, 6.2% for control of BRD at initial processing, and 3.0% for individual animal treatments. Specific macrolide antimicrobials used for control of BRD and individual animal treatment consisted of tulathromycin, gamithromycin, tildipirosin, and tilmicosin.

**Table 3:** Medically important regimens by antimicrobial class within use category (2018 and 2019 combined).

**Use category**	**Antimicrobial class**	**Number of feedyards**	**Total regimens all years**	**Percent of total regimens**	**Percent of total regimens by use category**
In-feed	Macrolide	16	1,128,515	72.76	77.76
	Tetracycline	9	77,574	5.00	
Control of bovine respiratory disease	Cephalosporin	8	51,777	3.34	12.67
	Fluoroquinolone	2	429	0.03	
	Macrolide	16	96,132	6.20	
	Penicillin	4	6,639	0.43	
	Phenicol	2	124	0.01	
	Sulfonamide	1	195	0.01	
	Tetracycline	10	41,305	2.66	
Individual animal treatment	Aminoglycoside	1	1,714	0.11	9.57
	Cephalosporin	18	14,355	0.93	
	Fluoroquinolone	17	27,934	1.80	
	Macrolide	20	46,654	3.01	
	Penicillin	6	3,146	0.20	
	Phenicol	19	30,725	1.98	
	Sulfonamide	8	4,269	0.28	
	Tetracycline	17	19,616	1.26	
Total			1,551,104	100.00	100

The next largest proportion of total regimens was the tetracycline class at 8.9%, this total value being comprised of 5.0% for treatment or control of BRD with chlortetracycline in the feed, 2.7% for control of BRD at processing with oxytetracycline, and 1.3% for individual animal treatment with oxytetracycline. Together, the uses of macrolide and tetracycline antimicrobials comprised 90.9% of all regimens. Fluoroquinolones and cephalosporins were used to a lesser extent, accounting for 1.8 and 4.3% of total regimens, respectively.

**Figure 1:**
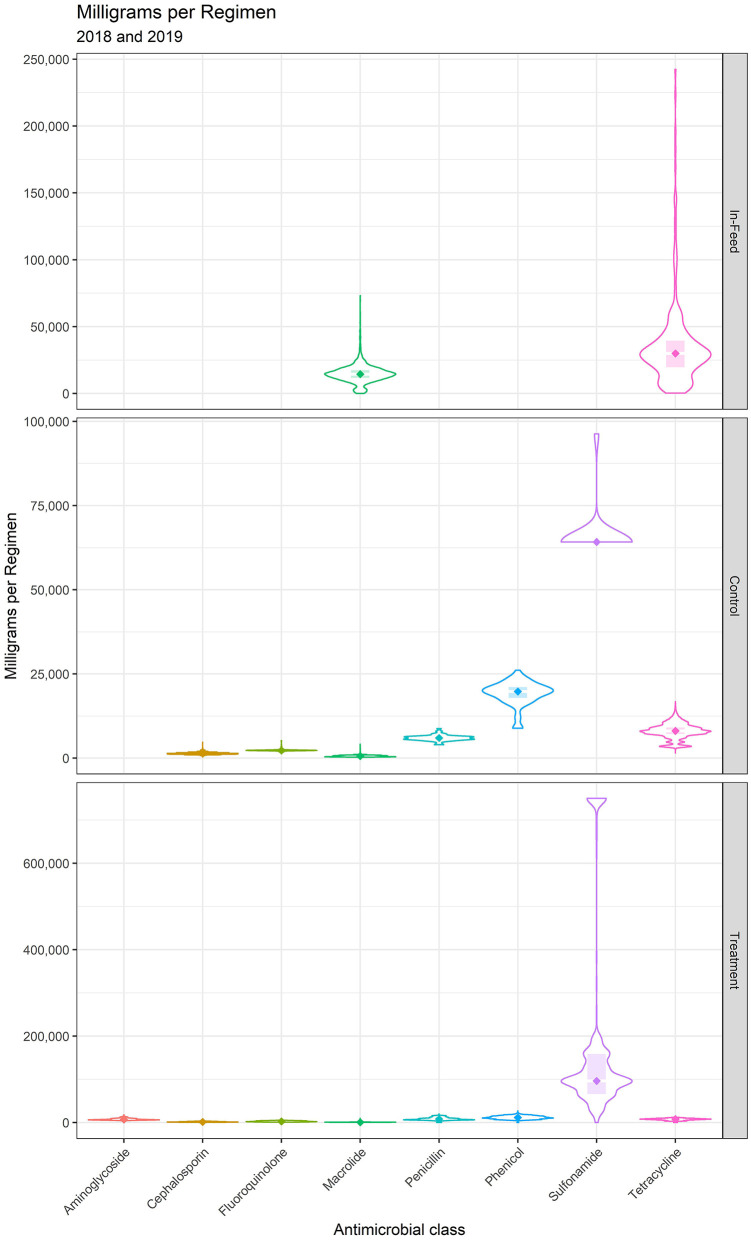
Milligrams per regimen for medically important antimicrobials.

**Figure 2:**
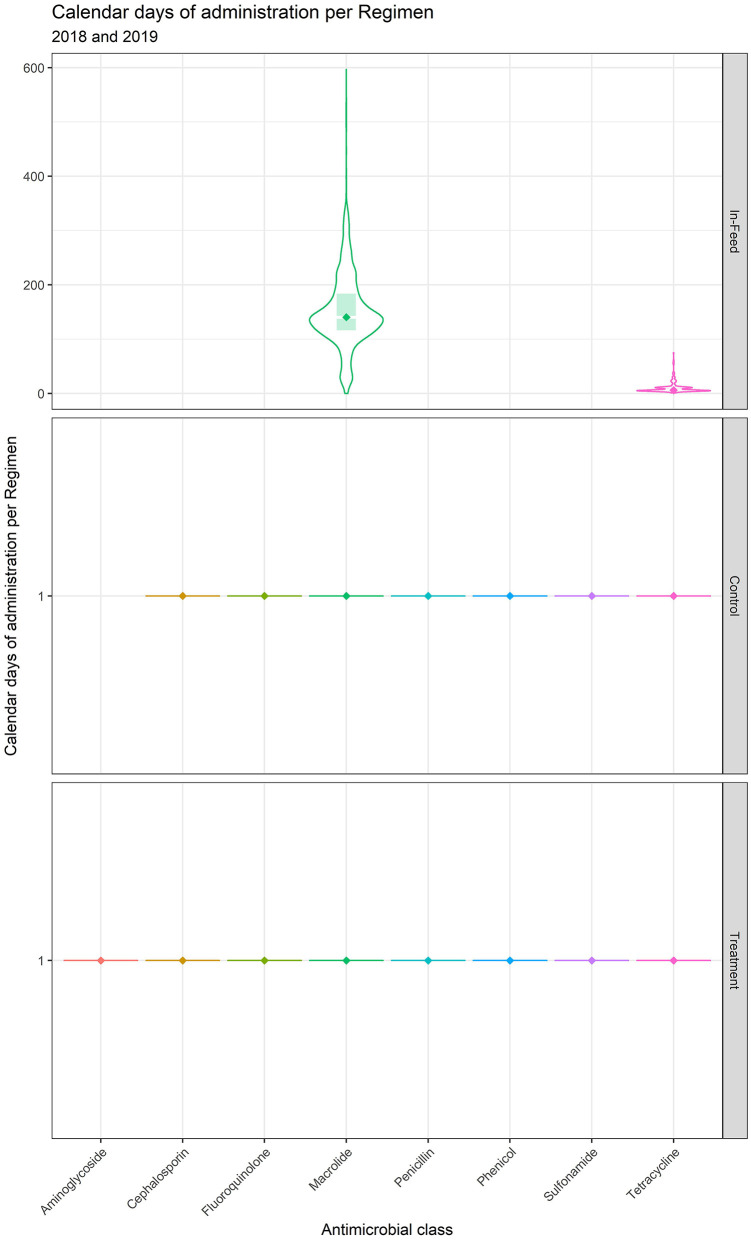
Calendar days of administration per regimen for medically important antimicrobials.

Regimens are described by milligrams of antimicrobial per regimen (mg/Reg) and calendar days of administration (CDoA/Reg) in Table 4 and Figures 1, 2. These regimen descriptions are derived from the 14 feedyards where regimens were directly described by granular data. Note that one calendar day of administration per regimen dominates for control of BRD and individual animal therapy, as these indications are largely administered as single-injection products. The feeding of a macrolide (tylosin) for reduction in the incidence of liver abscesses displays the highest CDoA/Reg but not the highest mg/Reg due to the label daily dose of 60–90 mg/head per day. The highest mg/Reg is displayed by the sulfonamide class of antimicrobials due to a high mg/kg dose, especially for the oral bolus formulation. The second highest mg/Reg value is for in-feed administration of a tetracycline (chlortetracycline) for treatment of BRD. There are two specific indications represented in the in-feed tetracycline data. The majority (90.2%) of the regimens were for the chlortetracycline indication for 10 mg/lb (22 mg/kg) bodyweight per day for up to 5 days for treatment of BRD. As an example, a 600 lb (273 kg) calf receiving 5 days of treatment at this dose would receive 30,000 mg in the regimen. In contrast, 9.8% of the in-feed chlortetracycline regimens were the 350 mg/head per day indication for control of BRD, with no defined duration of administration. A beef animal receiving this regimen for a theoretical period of 14 days would be recorded as 14 calendar days of administration with 4,900 milligrams for the regimen. The effects of these two very different regimens within the same class and use category may be observed in the relatively large variation around both CDoA and mg/Reg for chlortetracycline in Table 4.

**Table 4:** Regimens described as milligrams of drug per regimen and calendar days of administration per regimen grouped by antimicrobial class within use category.

		**Milligrams per regimen**			**Calendar days of administration**		
**Use category**	**Antimicrobial class**	**Median**	**Mean**	**Standard deviation**	**Median**	**Mean**	**Standard deviation**
In-feed	Macrolide	12,994	12,734	5,990	148	154	58
	Tetracycline	30,000	36,089	34,053	6	10	10
Control of BRD	Cephalosporin	1,400	1,429	342	1	1	0
	Fluoroquinolone	2,300	2,324	316	1	1	0
	Macrolide	600	627	246	1	1	0
	Penicillin	6,000	6,010	857	1	1	0
	Phenicol	19,800	19,162	3,630	1	1	0
	Sulfonamide	64,200	65,983	7,457	1	1	0
	Tetracycline	8,100	8,081	2,248	1	1	0
Individual animal treatment	Aminoglycoside	7,000	7,665	2,169	1	1	0
	Cephalosporin	1,600	1,749	821	1	1	0
	Fluoroquinolone	2,700	2,899	1,157	1	1	0
	Macrolide	900	1,091	801	1	1	0
	Penicillin	8,000	8,893	3,153	1	1	0
	Phenicol	11,700	11,913	3,685	1	1	0
	Sulfonamide	96,300	95,962	51,872	1	1	0
	Tetracycline	8,000	7,636	2,143	1	1	0

**Figure 3:**
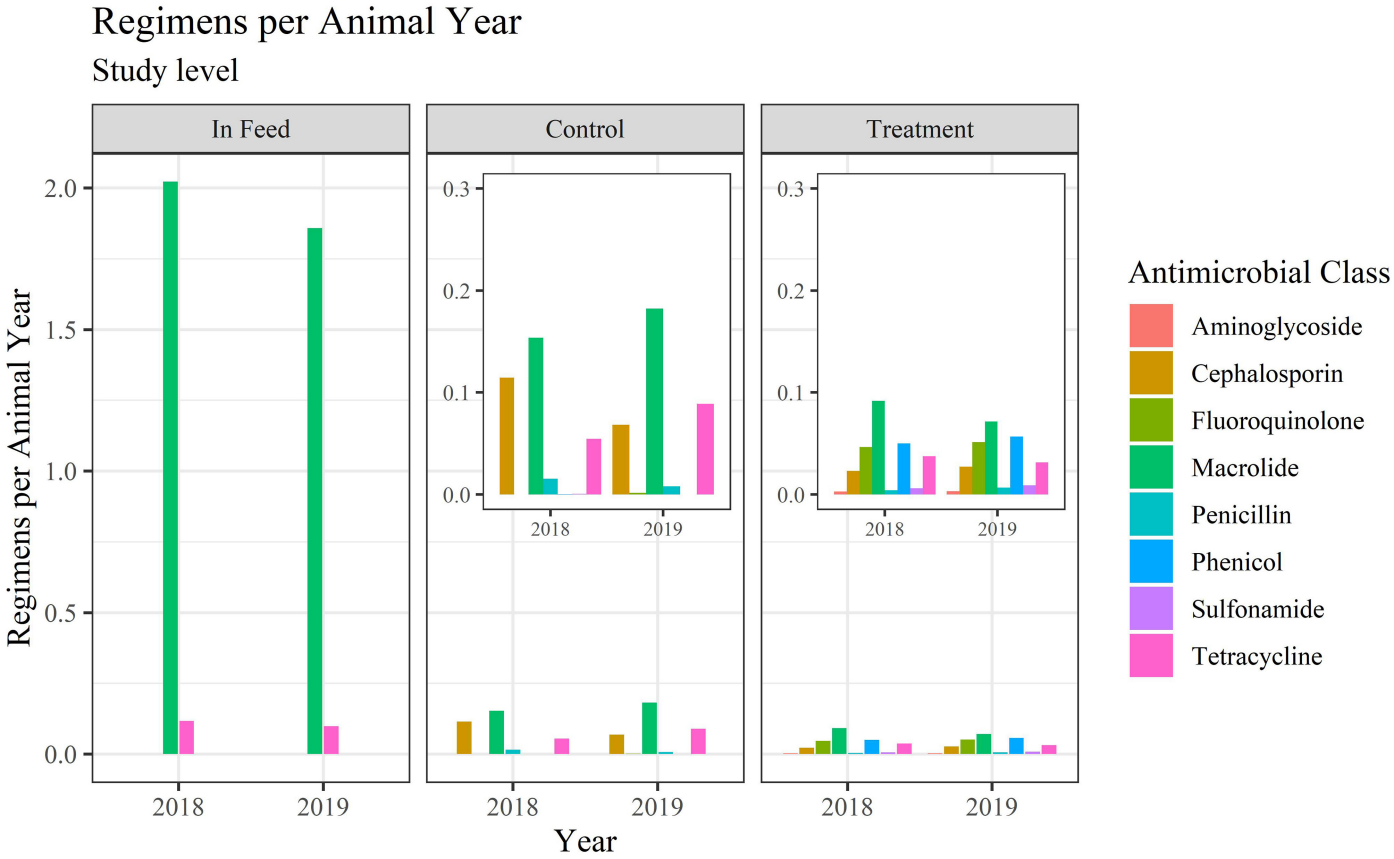
Regimens per animal year for medically important antimicrobials at the study level.

**Figure 4:**
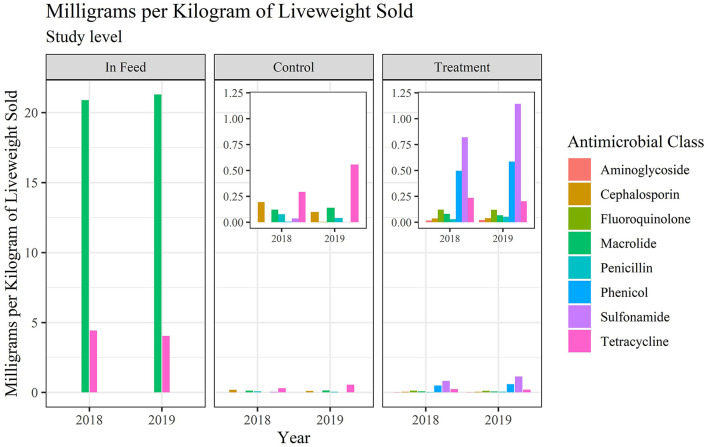
Milligrams per kilogram-LW for medically important antimicrobials at the study level.

Values, and percentage of total values, for Reg/AY and mg/kg-LW by antimicrobial class within use category are presented in Tables 5, 6, respectively. The values in Table 5 are presented in Figures 3, 4 for Reg/AY and mg/kg-LW, respectively. From both the tables and the figures it is apparent that by either metric the in-feed macrolides represent the highest values.

**Table 5:** Medically important Reg/AY and mg/kg-LW by antimicrobial class at the study level with feedyard count, expressed as values.

**Use category**	**Antimicrobial class**	**Reg/AY**		**mg/kg-LW**		**# Feedyards**	
		**2018**	**2019**	**2018**	**2019**	**2018**	**2019**
in-feed	Macrolide	2.02	1.86	20.90	21.29	16	15
	Tetracycline	0.12	0.10	4.42	4.04	9	6
	Total in-feed	2.14	1.96	25.32	25.33	–	–
Control of BRD	Cephalosporin	0.11	0.07	0.19	0.10	7	7
	Fluoroquinolone	0.0001	0.001	0.0003	0.003	1	2
	Macrolide	0.15	0.18	0.12	0.14	12	15
	Penicillin	0.02	0.01	0.08	0.04	3	3
	Phenicol	0.0004	0.00	0.006	0.00	2	0
	Sulfonamide^*^	0.0007	<0.0000	0.037	0.002	1	1
	Tetracycline	0.05	0.09	0.29	0.56	6	8
	Total control	0.34	0.35	0.73	0.84	–	–
Individual animal treatment	Aminoglycoside	0.00	0.00	0.02	0.02	1	1
	Cephalosporin	0.02	0.03	0.04	0.04	17	18
	Fluoroquinolone	0.05	0.05	0.12	0.12	17	16
	Macrolide	0.09	0.07	0.08	0.07	20	20
	Penicillin	0.004	0.01	0.03	0.05	5	6
	Phenicol	0.05	0.06	0.50	0.58	19	19
	Sulfonamide	0.01	0.01	0.82	1.14	8	6
	Tetracycline	0.04	0.03	0.23	0.20	16	17
	Total Individual	0.26	0.26	1.84	2.23	–	–
All uses	Overall total	2.74	2.56	27.89	28.40	–	–

Decimal places were increased up to 4 places to display a non-zero value where possible. ^*^Six regimens were reported for sulfonamides for control of BRD in 2019.

**Table 6:** Medically important Reg/AY and mg/kg-LW by antimicrobial class at the study level with feedyard count, expressed as percentages of the total value for that column.

**Use category**	**Antimicrobial class**	**Reg/AY (%)**		**mg/kg-LW (%)**		**# Feedyards**	
		**2018**	**2019**	**2018**	**2019**	**2018**	**2019**
In-feed	Macrolide	73.8	72.5	74.9	75.0	16	15
	Tetracycline	4.3	3.8	15.9	14.2	9	6
	Total % in-feed	78.1	76.4	90.8	89.2	–	–
Control of BRD	Cephalosporin	4.2	2.7	0.7	0.3	7	7
	Fluoroquinolone	0.004	0.1	0.001	0.01	1	2
	Macrolide	5.6	7.1	0.4	0.5	12	15
	Penicillin	0.6	0.3	0.3	0.1	3	3
	Phenicol	0.02	0.0	0.02	0.0	2	0
	Sulfonamide^*^	0.03	<0.000	0.1	0.005	1	1
	Tetracycline	2.0	3.5	1.1	2.0	6	8
	Total % control	12.4	13.6	2.6	3.0	–	–
Individual animal treatment	Aminoglycoside	0.1	0.1	0.1	0.1	1	1
	Cephalosporin	0.8	1.1	0.1	0.1	17	18
	Fluoroquinolone	1.7	2.0	0.4	0.4	17	16
	Macrolide	3.4	2.8	0.3	0.2	20	20
	Penicillin	0.2	0.3	0.1	0.2	5	6
	Phenicol	1.8	2.2	1.8	2.1	19	19
	Sulfonamide	0.2	0.3	2.9	4.0	8	6
	Tetracycline	1.4	1.2	0.8	0.7	16	17
	Total % Individual	9.5	10.0	6.6	7.9	–	–
All uses	Overall total	100.0	100.0	100.0	100.0	–	–

The total within each use category is the percentage of use for that use category (e.g., 78.07 % of total Reg/AY for 2018 was comprised of the in-feed use category, with 73.79% attributed to the macrolide class and 4.28% attributed to the tetracycline class). Decimal places were increased up to 3 places to display a non-zero value where possible. ^*^Six regimens were reported for sulfonamides for control of BRD in 2019.

### 3.2. Medically important antimicrobials at the feedyard level

A comparison of study level and feedyard level values for each antimicrobial class are presented in Table 7. Note that the feedyard level values are only for those feedyards reporting use of a specific antimicrobial, in contrast to the study level values calculated as total milligrams or regimens divided by total kg liveweight sold summed across all lots or animal years summed across all lots, respectively.

**Table 7:** Milligrams per kilogram-LW and Reg/AY calculated at the feedyard level by medically important antimicrobial class.

**Metric**	**Antimicrobial class**	**Number of feedyards**	**Year**	**Study level values**	**Feedyard level values**		
					**Mean**	**SD**	**Median**
mg/kg-LW	Aminoglycoside	1	2018	0.016	0.418	–	0.418
			2019	0.022	0.396	–	0.396
	Cephalosporin	18	2018	0.23	0.242	0.425	0.058
			2019	0.138	0.137	0.213	0.044
	Fluoroquinolone	17	2018	0.122	0.157	0.131	0.116
			2019	0.122	0.245	0.426	0.079
	Macrolide	20	2018	21.101	21.675	18.864	21.908
			2019	21.503	18.89	15.283	18.116
	Penicillin	6	2018	0.106	0.293	0.41	0.042
			2019	0.094	0.517	0.859	0.17
	Phenicol	19	2018	0.502	0.653	0.897	0.366
			2019	0.585	0.719	0.768	0.255
	Sulfonamide	8	2018	0.857	1.571	2.42	0.507
			2019	1.144	5.804	10.923	1.343
	Tetracycline	17	2018	4.952	42.061	92.672	2.425
			2019	4.795	33.448	94.161	1.359
Reg/AY	Aminoglycoside	1	2018	0.003	0.05	–	0.05
			2019	0.003	0.044	–	0.044
	Cephalosporin	18	2018	0.137	0.139	0.272	0.032
			2019	0.095	0.082	0.126	0.021
	Fluoroquinolone	17	2018	0.047	0.056	0.053	0.044
			2019	0.053	0.08	0.13	0.028
	Macrolide	20	2018	2.268	2.008	1.211	2.313
			2019	2.112	1.775	1.219	1.91
	Penicillin	6	2018	0.02	0.051	0.067	0.006
			2019	0.015	0.074	0.128	0.026
	Phenicol	19	2018	0.05	0.061	0.084	0.034
			2019	0.057	0.064	0.068	0.029
	Sulfonamide	8	2018	0.007	0.016	0.03	0.005
			2019	0.009	0.076	0.162	0.012
	Tetracycline	17	2018	0.209	1.061	2.549	0.147
			2019	0.219	0.936	2.908	0.156

### 3.3. Medically important antimicrobials categorized by individual animal treatment indication

Categorization by indication represents data from a 14-feedyard subset for which antimicrobial use data were directly associated with an indication and the regimens were not constructs. Regimen constructs for the other 6 feedyards occurred when total amount of drug could be assigned to a feedyard population, but the number of regimens had to be calculated by assigning a defined course dose (usually based on label dose and an estimated weight). In some cases, regimen constructs also occurred when a mg/head per day target dose was given for an in-feed antimicrobial and was assigned to a stated number of days in a defined population. Regimens per 100 head-in are reported for these 14 feedyards by disease indication associated with the regimens in Table 8. In Table 9, values are reported for the BRD indication stratified by use category, highlighting the ways in which antimicrobials are used to address BRD. The total regimens/head-in at the study level for each year in Table 9 sum to the value reported for BRD for that year in Table 8. In Tables 8, 9, feedyard level values are calculated using only data from the feedyards reporting use and do not include counts of head-in from non-reporting feedyards in the denominators.

**Table 8:** Medically important antimicrobial regimens per 100 head-in by indication at the study and feedyard level.

**Indication**	**Regimens per 100 head-in**						
	**Year**	**Study level**	**Feedyard level**				**Feedyard count**
			**Mean**	**SD**	**Median**	**cv (%)**	
Bovine respiratory disease	2018	37.1	39.6	37.1	26.2	93.7	14
	2019	36.2	40.5	49.7	29.0	122.7	14
Lameness	2018	0.8	1.0	1.8	0.3	174.2	13
	2019	0.9	0.8	0.8	0.4	104.0	13
Liver abscess control	2018	98.4	95.7	27.2	100.0	28.5	13
	2019	93.2	93.5	14.6	96.2	15.6	12
Other	2018	0.7	0.5	0.4	0.4	85.3	14
	2019	0.8	0.6	0.5	0.5	86.4	13

Note the change to Reg/100 head-in from Reg/AY in Table 7.

**Table 9:** Medically important antimicrobial regimens per 100 head in for bovine respiratory disease allocated to in-feed (chlortetracycline), control of bovine respiratory disease at processing, and individual animal treatment.

**Use category**	**Regimens per 100 head in**						
	**Year**	**Study level**	**Feedyard level**				**Feedyard count**
			**Mean**	**SD**	**Median**	**cv (%)**	
In-feed	2018	4.6	13.4	10.9	9.6	81.3	5
	2019	5.0	23.2	15.9	23.0	68.4	3
Control	2018	19.6	21.0	23.5	9.5	112	14
	2019	19.2	20.9	30.6	8.8	145.9	14
Individual treatment	2018	12.9	13.8	9.9	11.6	71.4	14
	2019	12.0	14.6	13.5	11.6	92.3	14

Note the change to Reg/100 head-in from Reg/AY in Table 8.

### 3.4. Individual animal treatment outcomes

Ten feedyards provided data of sufficient granularity such that outcomes of individual animal treatment for BRD, lameness, and all other categories combined (other) could be determined as reported in Table 10. In these 10 feedyards, BRD cases totaled 32,514 in 2018 and 26,359 in 2019. Lameness cases for 2018 and 2019 were 2,200 and 2,642, respectively. All cases in the other category of individual animal treatment (e.g., central nervous system disease, cellulitis) consisted of 4,666 cases in 2018 and 4,858 in 2019.

**Table 10:** Day 30 outcomes after individual animal treatment for bovine respiratory disease, lameness, and other indications.

**Disease**	**Percent of first treatments**						
	**Year**	**Outcome (day 30)**	**Study level (%)**	**Feedyard level**			**Feedyard count**
				**Median (%)**	**Mean (%)**	**Std dev (%)**	
Bovine respiratory disease	2018	Died	6.5	7.7	7.5	1.9	10
		Retreat	17.0	17.0	16.8	8.4	10
		Success	76.5	77.3	75.7	8.2	10
	2019	Died	7.3	8.7	9.2	3.9	10
		Retreat	15.3	13.9	15.0	7.0	10
		Success	77.4	76.4	75.9	8.0	10
Lame	2018	Died	5.4	5.1	6.8	5.8	8
		Retreat	8.1	8.2	8.9	5.5	8
		Success	86.5	87.4	86.1	9.3	9
	2019	Died	4.5	4.1	5.3	3.3	9
		Retreat	7.1	6.7	8.1	5.4	8
		Success	88.4	88.2	87.5	6.6	9
Other	2018	Died	4.5	7.5	12.7	13.2	9
		Retreat	12.5	14.5	13.6	8.8	10
		Success	83.0	79.1	74.9	10.9	10
	2019	Died	5.6	11.8	12.0	8.6	10
		Retreat	8.4	7.6	7.4	2.9	7
		Success	86.1	84.7	82.8	7.3	10

Outcomes are classified as mortality within 30 days for any cause, the animal being retreated for that disease within 30 days, or as a treatment success if neither of those instances were recorded by 30 days after the initial treatment. An individual animal is represented in only one category. Percentages are calculated based on the number of first treatments. The study level values in Table 10 are calculated based only on these 10 feedyards and do not include any data for the 10 feedyards for which granularity was not sufficient to determine outcomes.

### 3.5. Not-medically important antimicrobials

There were two not-medically important antimicrobials fed to cattle in this study, the ionophores monensin and lasalocid. Lasalocid was primarily utilized early in the feeding period to allow the concurrent feeding of chlortetracycline, if necessary, as these two antimicrobials are approved to feed in combination. Typically, when lasalocid was fed in the initial feed period, a switch to monensin was completed by 30 days on feed. In other cases, monensin was the only ionophore fed. Tables 11, 12 display regimen descriptions and usage metrics for not-medically important antimicrobials.

**Table 11:** Not-medically antimicrobial regimen descriptions for calendar days of administration per regimen (CDoA/Reg) and milligrams of antimicrobial per regimen (mg/Reg).

**Metric**	**Ionophore**	**Median**	**Mean**	**SD**
CDoA/Reg	Lasalocid	19	25	24
	Monensin	158	168	63
mg/Reg	Lasalocid	3,773	5,783	7,010
	Monensin	63,565	63,950	27,866

**Table 12:** Total mg/kg-LW and Reg/AY of not-medically important antimicrobials at the study and feedyard levels.

**Metric**	**Feedyard count**	**Year**	**Study level**	**Feedyard level**		
				**Median**	**Mean**	**SD**
mg/kg-LW	20	2018	89.44	104.23	122.31	66.81
		2019	96.10	107.45	121.23	62.61
Reg/AY		2018	2.35	2.60	2.83	1.02
		2019	2.28	2.08	2.52	1.01

## 4. Discussion

The objective of this study was to continue an initial period of antimicrobial use reporting in a convenience sample of U.S. beef feedyards and to further characterize challenges and opportunities in feedyard antimicrobial use monitoring. In addition, this report includes analysis of the indications for antimicrobial use and outcomes related to these indications. The feedyards contributing to the results reported here were samples of convenience utilized with the intent of investigating approaches to data collection and analysis. The nuances of the different selected metrics have been previously discussed, including an example of the impact of days-on-feed on the relative association of mg/kg-LW and Reg/AY for tylosin in the feed (5). An in-depth comparison of different metrics using the data presented in this paper is presented in a companion paper (10).

In this paper, the primary data analysis was at the study level, representing the entire body of data. In some results sections, study- and feedyard-level values are presented to illustrate the impact larger feedyards have on study level data compared to when all feedyards have an equal effect on the calculation of means and median values (feedyard level). The conclusion from these different data presentations is that the level at which data are aggregated and reported can have substantial effects on final metrics and this effect should be considered in interpretation of antimicrobial use data.

### 4.1. Differences in data format and granularity among the participating feedyards

Differences in record system granularity were illustrated by only 14 of the 20 participating feedyards having data sufficient to describe antimicrobial regimens without the use of a calculation construct such as a defined daily dose or a defined course dose. When considering therapeutic outcomes, still fewer feedyards (10) had data sufficient for determining outcomes linked to a specific treatment indication; and among these feedyards not all feedyards had a documented cause of death associated with recorded mortalities. Also, challenges existed in the ability to associate each ration fed throughout the feeding period with a specific cohort of cattle and the exact inclusion amount for each ingredient within each of those rations. While the feedyards have these data within computerized systems, these systems are frequently different from their daily lot and treatment systems which introduces considerable challenges when tying them together.

These differences in granularity and data format are examples of challenges in data interoperability. While using the same electronic record system may be beneficial in the efficiency of data aggregation, this approach does not remove all interoperability challenges. The need to improve data interoperability has been identified as a major challenge to antimicrobial use reporting for both veterinary and human medicine antimicrobial use monitoring programs by the United States Presidential Advisory Council on Combating Antibiotic-Resistant Bacteria (PACCARB) (12).

### 4.2. Comparison to other North American feedyard antimicrobial use data

Other researchers have also reported on antimicrobial use in North American feedyards. Table 13 summarizes characteristics of 2 other publications along with this paper. These authors are appreciative of the detailed and transparent reporting of methods in these 2 other papers and the willingness of the first authors to clarify our understanding of the methods. We respectfully compare and contrast methods to illustrate the effects of different approaches within the North American feedyard environment. When comparing and contrasting methods and reported values, the terms “this paper”, “reported here” or “these authors” always refers to the current report/manuscript.

**Table 13:** Characteristics of 3 different papers quantifying antimicrobial use in North American feedyards.

	**Brault et al**.	**Rutten-Ramos et al**.	**Apley et al**.
Metrics reported	nADD of active antimicrobial ingredient/100,000 cattle	Milligrams of active antimicrobial ingredient/kg liveweight sold	Milligrams of active antimicrobial ingredient/kg of liveweight sold and Regimens/Animal Year, with regimens described by mg/Reg and Calendar Days of Administration (CDoA)/Reg
Populations and antimicrobial attribution	Defined as cattle lots received in the designated study period with all drugs administered (characterized by administration records) attributed to each lot during their entire feeding period.	Defined as cattle lots placed in the designated periods with all drugs administered (characterized by lot records of drug billing to each lot converted to active ingredient) attributed to each lot during their entire feeding period.	Defined as the cattle lots closed out (sold) in the study year with all drugs administered (characterized by administration records) and days on feed attributed to the study lots for the entire feeding period.
Assignment of nDDD to single injection antimicrobials	nDDD/administration and ADD (mg/kg per day) assigned values are included in supplemental information in the Brault, et al. article. An nDDD value of 3 DDD per administration was assigned to 8 injectable antimicrobials and one oral bolus antimicrobial. An nDDD value of 2 DDD per administration was assigned to two injectable products.	nDDD values per regimen or DDD values were not assigned. Analysis addressed mg of active product.	nDDD/administration and ADD (mg/kg per day) values are not assigned. Administered mg of active substance and regimens are reported along with descriptions of the regimens as CDoA/Reg and mg/Reg.
Assignment of nDDD to in-feed antimicrobials	ADD (mg/animal per day) assigned values are included in supplemental information for the Brault, et al. article. The DDD value for chlortetracycline in the feed is established by selecting a median dose from the mg/day regimens used for respiratory disease (5,500 mg/day). The administered daily dose is then divided by the DDD value to assign an nADD value to that administration. The DDD value for tylosin in the feed was established from the consideration of injectable doses (17.6 mg/kg for a 450 kg animal, 7,920 mg).	nDDD values per regimen or DDD values were not assigned. Analysis addressed mg of active product.	ADD (mg/animal per day) values are not assigned. Administered regimens and descriptions of the regimens as calendar days of administration (CDoA)/Reg and mg/Reg are reported. Mean mg/CDoA may be calculated from these values.

DDD, Defined Daily Dose; nDDD, number of Defined Daily Doses; ADD, Animal Daily Dose.

Rutten-Ramos et al. published a population-level analysis of antibiotic use and mortality in U.S. feedyards over a 10 year period from 2010 to 2019 (13). The authors reported use of mg of active ingredient over a denominator of kg liveweight sold. The analysis unit was the lot, where the numerator of milligrams of active antimicrobial product was calculated on economic record attribution of antimicrobials to each lot, expressed over the denominator of the total kilograms of cattle marketed from that lot. Although the overall total reported mg of active substance per kilogram of liveweight sold in the Rutten-Ramos et al., paper is similar to the value reported here, where the use of tylosin and chlortetracycline in the feed comprise the highest values, the relative contributions of tylosin and chlortetracycline differ substantially.

A report of antimicrobial use on 36 Western Canada beef feedlots was published by Brault et al. (14). The analysis unit remains the lot with administration records serving as the data source. Brault et al. captured the amount or administrations of active ingredient administered to each lot and then converted these amounts to the number of Animal Daily Doses (nADD). For individually administered antimicrobials (injectable or oral) an ADD_kg_ value was calculated for each antimicrobial. The nADD for each administration was then calculated by dividing the milligrams of antimicrobial administered by the product of the ADD_kg_ (mg/kg per day) multiplied by the weight of the animal. The calculated nADD per administration was 3 ADDs for 8 injectable antimicrobials and one oral antimicrobial, and 2 ADDs for two other injectable antimicrobials.

Brault et al. illustrate the variability in possible nADD values and for this reason these authors have not assigned a number of ADD to single injection antimicrobials (15). The only reason for the international emphasis on accurately evaluating antimicrobial use in feedyards and other food animal production facilities is as a multi-pronged approach to mitigating antimicrobial resistance. These authors strongly feel that assigning durations of therapy to single injection antimicrobials is fraught with the illusion of precision, and this concern about inaccuracy is amplified when these durations are interpreted as a proxy for periods of resistance selection pressure.

Brault et al. used a standard ADD for chlortetracycline in the feed (5,500 mg/head per day) calculated from the reported range for metaphylaxis or treatment of *Histophilus somni*. This ADD value was then divided into the total administered amount of chlortetracycline for each in-feed indication for an individual lot to assign an nADD value. It is important to recognize that extralabel use of medically important antimicrobials in the feed is allowed in Canada; veterinarians may prescribe off label regimens. This practice is illegal in the United States. The effects of this definition method on the reported nADD/100,000 head may be demonstrated. From percentages reported by Brault et al., it may be calculated that 52.5% of total in-feed use was chlortetracycline for the purpose of liver abscess control. The liver abscess control daily dose used for chlortetracycline was not reported and there is not a Canada-labeled dose for this indication which may be referenced. If the U.S. approved chlortetracycline dose is used as a proxy (70 mg/head per day), then use at this dose would mean that 78.5 days of feeding chlortetracycline for liver abscess control would be counted as one ADD of 5,500 mg. This situation likely contributed to in-feed antimicrobial nADD/head values (calculated by dividing nADD/100,000 head values by 100,000) for in-feed antimicrobial use of only 11.57, 11.25, 10.95, and 9.92 for study periods 1–4, respectively, even though daily administration of chlortetracycline for liver abscess control was a common practice. A similar approach was taken for calculating tylosin nADD for liver abscess control, where the ADD used for calculation of in feed nADD was determined from an injectable tylosin regimen with much higher daily milligram values.

These authors' purpose in comparing and contrasting different approaches to feedyard data analysis is to further emphasize points made by Brault et al. in relation to the effect of calculation decisions related to injectable antimicrobials; these points are the need for complete transparency in data collection and analysis techniques, and that methods really matter in how data are interpreted (15). The above example illustrates these points by illustrating the effects of what indications and regimens are included in analysis of an antimicrobial product (e.g., selection of ADD values for chlortetracycline in-feed administration for both respiratory disease and reduction in liver abscesses), and also emphasizes the need for extreme caution when comparing antimicrobial use estimates from different populations (or manuscripts) with different calculation methods.

Sales of medically important antimicrobials for use in food-producing animals in the United States are reported annually by the Food and Drug Administration Center for Veterinary Medicine (16). While the data collection methods used in the annual FDA report are different from the methods used here, the dominance of macrolide and tetracycline class antimicrobial sales attributed to cattle is in agreement with the data reported here and other published feedyard antimicrobial use data (13, 14).

### 4.3. Antimicrobial indications and use

In contrast to the authors previous publication of 2016–2017 feedyard antimicrobial use data, in this paper the reporting of indication-specific antimicrobial use values and antimicrobial therapeutic outcomes have been added. The authors feel that recognizing the causative disease challenges for antimicrobial use and characterizing the results of addressing these diseases with antimicrobials are fundamental components of antimicrobial stewardship. It is not proposed here that the reported therapeutic outcomes are indicative of the specific efficacy of the antimicrobials in the therapy of these diseases, rather they are grossly representative of the aftermath of the antimicrobial-disease interactions in these specific systems.

### 4.4. Comparison of antimicrobial therapy outcomes to other papers

#### 4.4.1. Bovine respiratory disease

Respiratory disease treatment outcomes have been summarized in a systematic literature review by DeDonder and Apley (17). In the evaluation of 31 randomized, controlled, prospective clinical trials, the median clinical success rate (not treated again for BRD by study conclusion) was 71%. The duration of these clinical trials ranged from 10 to 28 days with one additional outlier of 45 days. In the study reported here, the observed BRD success rates at 30 days were 76.5 and 77.4% for 2018 and 2019, respectively, similar to the median value for the systematic literature review. However, the cattle populations in the DeDonder and Apley review were primarily calves classified as being at a high risk for BRD; the population in the study reported here consisted of all cattle in the study population.

The systematic review by DeDonder and Apley also evaluated the case fatality rate in 25 of the clinical trials which reported mortality in the treated animals. The median case fatality rate in these clinical trials was 1% when calculated only for mortality confirmed as due to BRD on necropsy. In the data reported here (10), the 30 day mortality rates from all causes for cattle treated for BRD were higher, at 6.5 and 7.3% for 2018 and 2019, respectively. Differences may be due to the clinical trials reporting mortalities for shorter periods after treatment, allowing less time for treatment failures to transition to mortalities. Also, the data reported here include all-cause mortality within 30 days of treatment as some data sets did not assign cause of death to mortalities; this difference is probably minimal as the agreement between BRD treatment and death due to BRD being confirmed at necropsy has been demonstrated to be very high (18).

#### 4.4.2. Lameness

Terrell et al. evaluated lameness cases and outcomes in 6 large commercial feedyards in Kansas and Nebraska (19). Of 2,532 lameness cases, comprised of 9 different diagnostic categories, 1,735 (68.5%) shipped to slaughter with their unaffected pen mates, 230 (9.1%) were shipped to slaughter ahead of their pen mates for salvage, and 567 cases (22.4%) died prior to being sold. The mean (±standard deviation) interval between diagnosis and death ranged from 10 ± 16 days for upper limb lameness to 29 ± 35 days and 29 ± 29 days for interdigital phlegmon (foot rot) and laceration of the foot or hoof wall, respectively. In the study reported here, the death loss by 30 days post-first treatment for a condition recorded as lameness was 5.4 and 4.5% for 2018 and 2019, respectively. The differences in mortality rates may be due to both differences in populations and different duration of the periods at risk for mortality due to lameness or associated diseases.

### 4.5. Implications for antimicrobial stewardship

This report again emphasizes that the largest uses of antimicrobials in the participating feedyards are tylosin and chlortetracycline in the feed, related to disease indications of liver abscesses and bovine respiratory disease, respectively. The results indicate that different antimicrobial use metrics may illustrate the same major trends, but that understanding how each specific metric is derived is vital to interpretation. If benchmarking is performed in the future, issues of standardization and interpretation must be addressed for these systems to have utility. The evaluation of the utility of the information presented here and by others should be led by those making the daily antimicrobial use decisions at the producer-veterinarian level.

Despite the challenges in reaching agreement on antimicrobial use monitoring standards, data such as presented here are a crucial component in global efforts to mitigate antimicrobial resistance through first understanding our current uses.

## Data availability statement

The datasets presented in this article are not readily available because a condition of participation by the participating beef feedyards was anonymity of both the participants and the original data. For this reason, the raw data are not available. Requests to access the datasets should be directed to mapley@vet.k-state.edu.

## Author contributions

MA recruited the feedyards, coordinated the data collection, and wrote the manuscript drafts. NS and DA conducted the data analysis, wrote the R code, and contributed to the paper content. BL and RS contributed to the project direction from conception, participated in determination of analytical methods and quality assurance, evaluated clarity of data presentation, and commented on manuscript drafts. All authors contributed to the article and approved the submitted version.
